# Choroid vascular changes in hyperopic anisometropia amblyopia using SS-OCTA

**DOI:** 10.1186/s12886-023-03121-x

**Published:** 2023-09-18

**Authors:** Yiwen Cao, Yadi Zhang, Xiaopeng Gu, Dehai Zhu, Liu Yang

**Affiliations:** 1 Department of Pediatric Ophthalmology, Peking University First Hospital, No. 8 Xishiku Street, Xicheng District, Beijing, 100034 China; 2 Department of Ophthalmology, Peking University First Hospital, No. 8 Xishiku Street, Xicheng District, Beijing, 100034 China

**Keywords:** Amblyopia, Hyperopia, Anisometropia, OCTA, Choroid, Choroidal thickness, Choroidal capillary, Choroidal vascularity volume, Choroidal vascularity index

## Abstract

**Purpose:**

To observe and understand the structural changes in choroidal vessels in eyes with hyperopic anisometropic amblyopia using swept-source optical coherence tomography angiography (SS-OCTA).

**Methods:**

A total of 44 patients were enrolled in this study: 22 children with hyperopic anisometropic amblyopia and 22 age-matched controls. SS-OCTA was used to scan the 6*6 mm macular area of their eyes. The average choroidal thickness (CT) and choroidal capillary flow area (CC) in a 3 mm diameter area centered on the macular area were obtained. The choroidal vascularity volume (CVV) was automatically extracted and 3D reconstructed by inbuild software, and the three-dimensional choroidal vascularity index (3D-CVI) was calculated. The effect of amblyopia on the choroidal vessel structure was assessed using generalized linear estimating equations (GEEs) corrected for axial length, sex, age, and best-corrected visual acuity.

**Results:**

The CC was greater in amblyopic eyes than in fellow eyes (*P* = 0.014) but was not significantly different from that in control eyes (*P* = 0.963). After correcting for sex, age, axial length, and visual acuity using GEEs, the mean CT in the amblyopic eyes was greater than that in the fellow eyes (*P* = 0.030) but was not significantly different from that in the control eyes (*P* = 0.160). The 3D-CVI in amblyopic eyes was higher than that in control eyes (*P* = 0.038) but was not significantly different from that in fellow eyes (*P* = 0.407). The three-dimensional choroidal vascularity volume (3D-CVV) was higher in amblyopic eyes than in fellow eyes (*P* = 0.046) and control eyes (*P* = 0.023).

**Conclusions:**

We found that eyes with hyperopic anisometropic amblyopia demonstrated higher CT, CC and 3D-CVV values than the contralateral eyes after correction, while the 3D-CVI was unchanged. Compared with control eyes, amblyopic eyes had higher 3D-CVV and 3D-CVI values but similar CT and CC values. Amblyopic eyes may have different choroidal vascular structures from fellow and control eyes.

## Introduction

Amblyopia is one of the leading causes of visual impairment in preschool children in China [1, 2], and untreated amblyopia can impact the academic lives of affected children [3]. According to previous studies, anisometropia is responsible for most cases of amblyopia [4]; this anisometropic amblyopia is caused by an abnormal interaction between the two eyes, resulting in amblyopia eyes having lower best-corrected visual acuities than the fellow eyes [5].

The amblyopic eye is usually considered free of ocular structural pathology. The normal structure and function of the choroid plays an important role in the nutrition and thermoregulation of the retina. In recent years, choroidal changes have been observed in a range of diseases, including amblyopia [6, 7]. Some studies have demonstrated thinner choroids in amblyopic eyes than in fellow and control eyes. However, studies investigating choroidal vascular structure changes have reached different conclusions [8, 9]. Furthermore, choroidal thickness and vascular structures are affected by a variety of factors, such as age, sex, axial length (AL), and refractive error [10–13]. AL and refractive error differences are common between amblyopic eyes and normal eyes, and ignoring these factors will affect the accuracy of the results. However, whether there are anatomical differences in the choroidal vascular structures between amblyopic and normal eyes remains to be understood.

With the development and widespread use of optical coherence tomography angiography (OCTA), it has become possible to understand the hierarchical structure of the retina and choroid in more detail. By using the variation in OCT signal caused by moving particles, such as red blood cells (RBCs), as the contrast mechanism for imaging blood flow, OCTA allows the visualization of functional blood vessels in the eye. In recent years, the use of swept-source OCTA (SS-OCTA) has become more beneficial for exploring deeper choroidal changes because of its faster scanning speed, clearer imaging quality, and greater scanning depth [14].

The choroidal vascularity index (CVI) is an OCT-based marker of choroidal vascularity that reflects the relationship between the choroidal vascular area and the total choroidal area. Previous studies have demonstrated differences in the CVI among patients with different refractive statuses [15], as well as amblyopia [16]. In previous studies, the CVI was typically obtained from a single B-scan image of the macula foveal region. Using one scan image covering the macula fovea to analyze changes in the CVI may not reveal the overall state of the choroidal vessels within a certain area. Choroidal vessel identification and 3D reconstruction can provide more information on the overall state of the choroid within a certain region and can provide a better understanding of the effect of disease on the choroidal vessels [13]. To our knowledge, there are no studies on the changes in 3D choroidal vessels between amblyopic and normal eyes.

In our research, we used SS-OCTA to scan the eyes of patients and age-matched normal controls and analyzed the differences in the mean choroidal thickness (CT), choroidal capillary flow area (CC), 3D-reconstructed choroidal vascular volume density (3D-CVI), and 3D-reconstructed choroidal vascular volume (3D-CVV) between amblyopic eyes, fellow eyes and control eyes. To assess and increase our understanding of the structural differences in the choroidal vasculature in the macula of hyperopic anisometropic amblyopia and clarify the effect of amblyopia on 3D choroidal vascular structure,

## Methods

### Demographic data

This was a retrospective study approved by the Peking University First Hospital Human Research Ethics Committee (2022Yan187). Data collection was performed in accordance with the Declaration of Helsinki.

The data were collected consecutively from February 2022 to June 2022. Children aged 4 to 12 years who attended the pediatric ophthalmology department of Peking University First Hospital and underwent OCTA examination to screen for hyperopic anisometropic amblyopia were initially included as the amblyopia group. The criteria for diagnosing hyperopic anisometropic amblyopia were determined according to the American Academy of Ophthalmology guidelines [17]. The inclusion criteria were as follows: 1, an equivalent spherical (SE) diopter (D) difference between both eyes > 1 D; 2, hyperopia in both eyes; and 3, two-line difference in best-corrected visual acuity (BCVA) between both eyes, with the more hyperopic eye having worse visual acuity. The exclusion criteria were as follows: 1, the presence of retinal or choroidal disease; 2, treatment with instruments that could potentially affect the retinal choroid; 3, esotropia or constant exotropia or vertical strabismus; 4, glaucoma; 5, previous internal eye surgery; 6, obstruction in the visual axis; 7, systematic disease; and 8, inability to cooperate with the examination for any reason.

We collected data from age-matched patients with mild hyperopia (< + 1.50 D), orthopia or mild myopia (> -0.50 D) who had no amblyopia and no ocular or systemic disease as the control group. All participants’ cycloplegia refraction, axial length, and BCVA values were also collected.

### Image acquisition

SS-OCTA (VG100; SVision Imaging, Ltd., Luoyang, China) was used to scan the macular area of the eyes of the participants. The scan was centered at a wavelength of approximately 1050 nm and performed at a rate of 100,000 A-scans per second. The maximum axial and estimated lateral resolutions in the tissue were approximately 5 μm and 15 μm, respectively, and the scanning depth was 3 mm. Choroidal vascular distribution and CT data were obtained by a raster scanning protocol with 1024 × 1024 B scans covering a 6 mm × 6 mm area centered on the central macular fovea. Eye-movement artifacts during and between scans were minimized by using the built-in eye-tracking mode of the device based on the integrated confocal scanning laser detector lens.

**Fig. 1:**
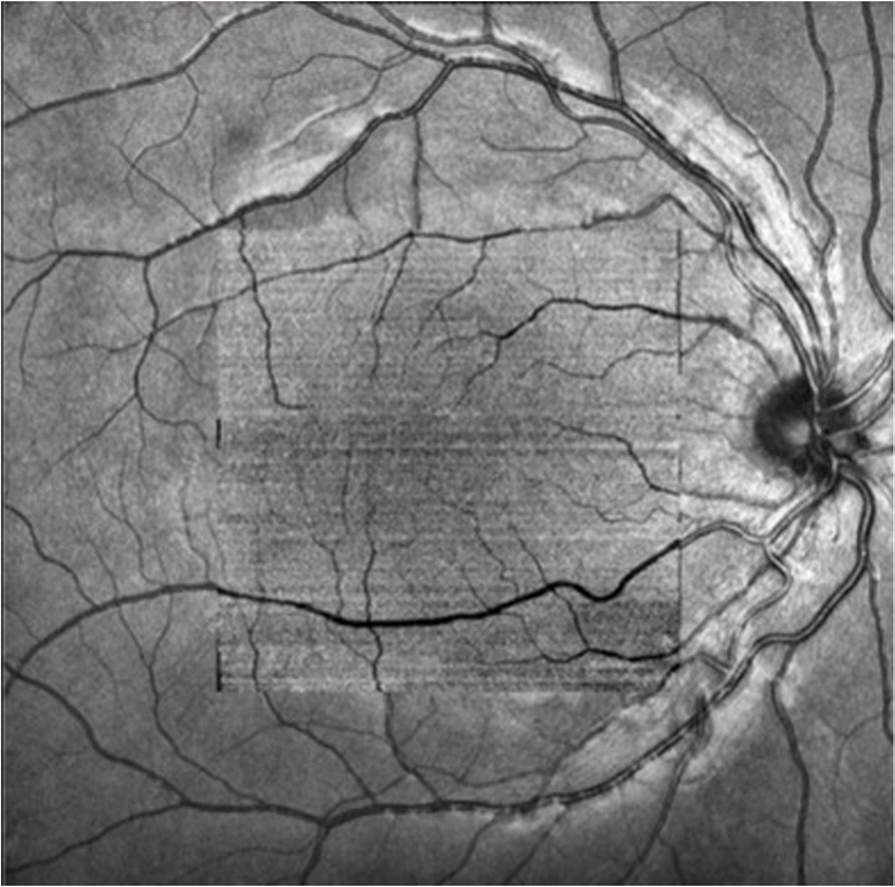
OCTA scan of 6 mm × 6 mm area centered on the macula fovea

**Fig. 2:**
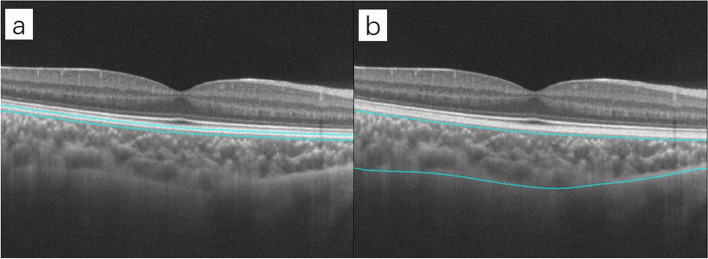
Definition of the choroid and choroidal capillaries. The inbuild software automatically performs layer segmentation. **a**. Choroid: the region spanning from the outer border of the retinal pigment epithelium-Bruch's membrane complex to the choroid-sclera junction. **b**. Choroidal capillaries: the area 10 μm above Bruch's membrane to 25 μm below it

Quantitative analysis was performed using a 6 mm × 6 mm OCTA scan (Fig. 1). When performing the examination, the software automatically scores the quality of the scan (1–10, with 10 being the highest score), and if the score for that scan is below 7, the scan will be performed again for the child. The scan with the highest quality was selected for analysis. Choroidal parameters were analyzed using built-in software (V 1.36.10), and all subjects' axial lengths were entered for image magnification correction. The software automatically performs layer segmentation and quantitative analysis [18] (Fig. 2). Data were reviewed by the researcher before extraction, and if there were errors in the stratification, manual changes were made to the segmentation to ensure accuracy. To avoid incomplete areas due to the magnification effect caused by hyperopia and the effect of retinal macrovascular occlusion, a circular area with a diameter of 3 mm centered on the central macular fovea was selected for analysis. To avoid interference of the circadian rhythm on the choroid, all subjects were examined from 9:00 am to 12:00 pm.

The choroid was defined as the area from the outer border of the retinal pigment epithelium-Bruch's membrane complex to the choroid-sclera junction, and the thickness was recorded as the average choroidal thickness in this area (Fig. 2). The software automatically extracted the image of the scanned choroidal region and calculated the average choroidal thickness in that region.

**Fig. 3:**
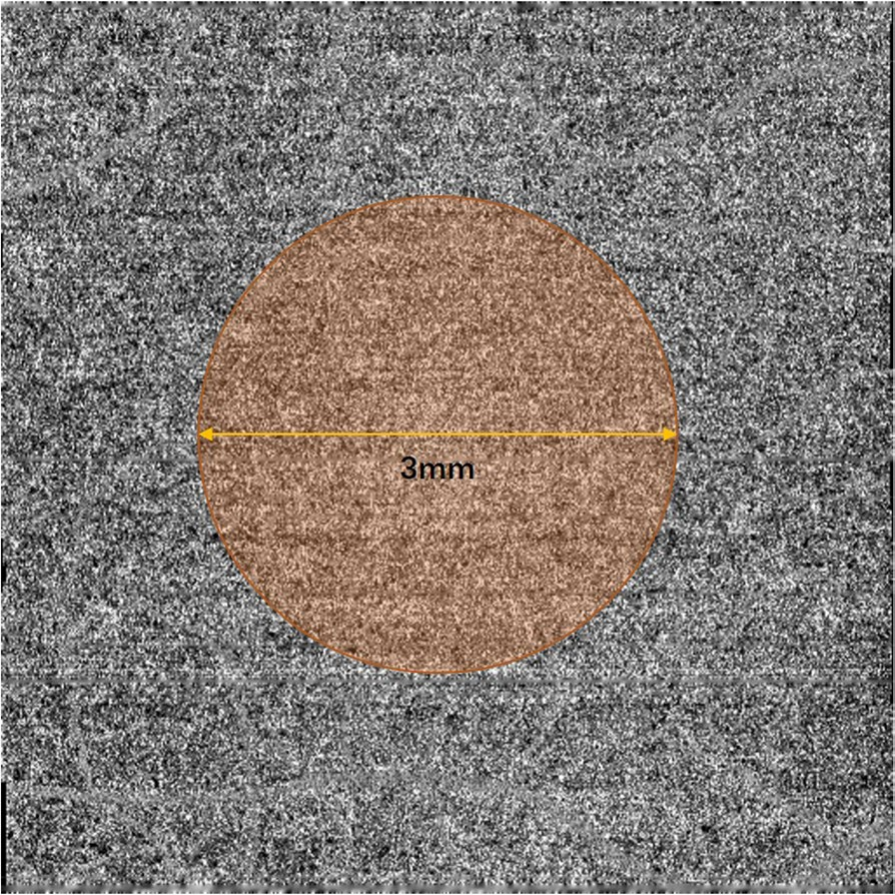
Automatic extraction and computational analysis of choroidal capillary images in a 3 mm-diameter circle centered on the fovea

The choroidal capillaries were defined as those in the area 10 μm above Bruch's membrane to 25 μm below it. The inbuild software also automatically identifies this region, acquires the en face image (Fig. 2), selects the choroidal capillaries using the software's threshold function and automatically calculates the flow area (Fig. 3).

**Fig. 4:**
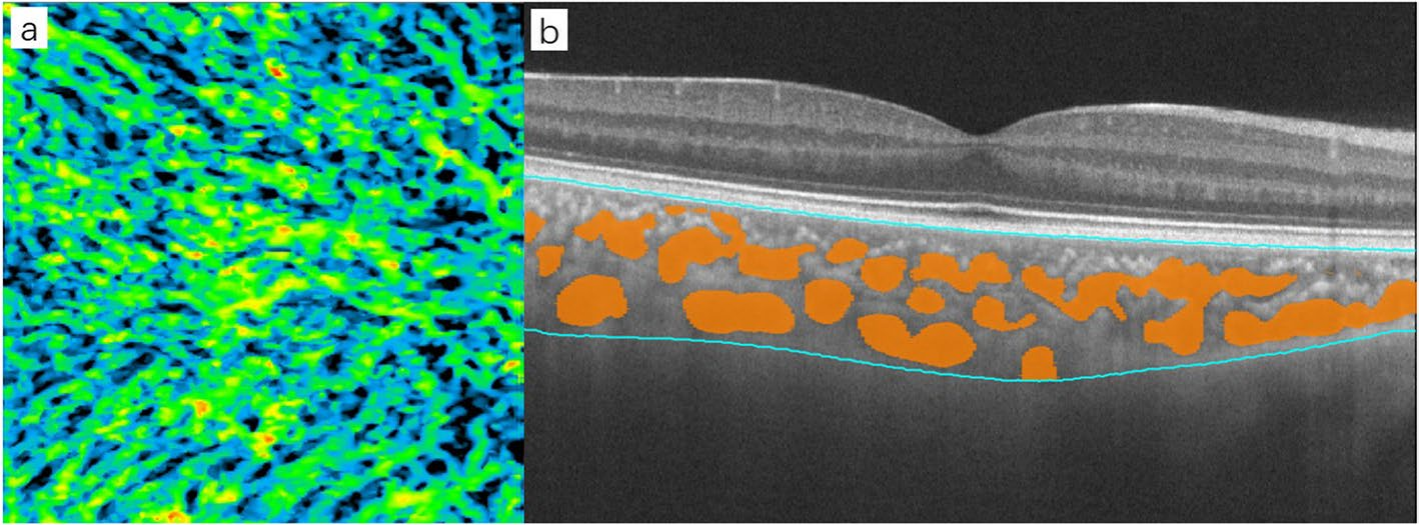
The software automatically extracts the scanned choroidal vessels and performs 3D reconstruction to calculate the CVV and CVI. **a** The software automatically extracts the choroidal large and medium vessels in each scan image. **b** 3D vessel reconstruction

The images of large and medium vessels were also automatically extracted by the software (Fig. 4), and 3D reconstruction was performed to calculate the choroidal large and medium vessel volume (3D-CVV). The 3D-CVI was defined as the ratio of the choroidal vessel volume to the total choroidal volume (TCV), which reflects the choroidal vascular volume in the Sattler.

### Statistical analyses

SPSS (version 26.0; IBM Corp., Armonk, NY, USA) was used for all statistical analyses. A chi-square test was used to compare the sex ratio between the two groups. The Kolmogorov–Smirnov test was used for normality testing. Normally distributed variables are expressed as the mean and standard deviation (mean ± SD), and nonnormally distributed variables are expressed as the median and interquartile range (IQR). The means of normally distributed quantitative variables were compared using a t test. For nonnormally distributed data, the Mann–Whitney U test was used for testing. The two eyes in the amblyopic group were compared using the paired test, and amblyopic eyes were compared with control eyes using the between-group test. Multivariable generalized estimating equation (GEE) models were used to adjust for the potential effects of axial length, sex and age on CT, 3D-CVI and 3D-CVV. *P* < 0.05 was considered to indicate statistical significance.

## Results

### Demographic data

A total of 44 children were included in this study, 22 with hyperopic anisometropic amblyopia and 22 in the control group. The clinical characteristics of each group are presented in Table 1. There were no significant differences between the amblyopic and normal control groups in terms of age or sex ratio. The mean axial length was 21.56 ± 1.06 mm, 22.26 ± 1.15 mm, and 22.96 ± 1.03 mm in the amblyopic, fellow, and control eyes, respectively, and the differences among them were significant (*P* < 0.001). The mean SE was + 5.09 ± 2.01 D, + 3.05 ± 1.98 D and + 0.25 ± 0.68 D in amblyopic, fellow and control eyes, respectively (*P* < 0.001). The mean logMAR BCVA was 0.343 ± 0.255, 0.104 ± 0.114 and 0.0029 ± 0.531 in the amblyopic, fellow and control eyes, respectively, and the differences among the groups were significant (*P* < 0.001).

**Table 1:** Clinical characteristics of the subjects

	AE mean ± SD	FE mean ± SD	CE mean ± SD	*P* value^*^	*P* value^#^
N	22	22	22		
Age (years)	6.54 ± 1.37	6.54 ± 1.37	6.63 ± 1.46	-	0.558
Sex					
Male	12 (52.4%)	12 (52.4%)	11 (50.0%)		0.763
Female	10 (47.6%)	10 (47.6%)	11 (50.0%)		
AL (mm)	21.56 ± 1.06	22.26 ± 1.15	22.96 ± 1.03	< 0.001	< 0.001
Diopter (D)	+ 5.09 ± 2.01	+ 3.05 ± 1.98	+ 0.25 ± 0.68	< 0.001	< 0.001
BCVA (logMAR)	0.343 ± 0.255	0.104 ± 0.114	0.0029 ± 0.531	< 0.001	< 0.001

*AE* amblyopic eye, *FE* fellow eye, *CE* control eye, *N* number, *BCVA* best-corrected visual acuity, *AL* axial length, *SE* spherical equivalent, *D* diopter, *SD* standard deviation

^*^Paired t test between amblyopic eyes and fellow eyes

^#^t test between amblyopic eyes and control eyes

### Choroidal capillary flow area (CC)

The CC did not conform to a normal distribution (*P* = 0.032, *P* = 0.003, and *P* = 0.015 for amblyopic, fellow and control eyes, respectively). The median CC was 6.69 (0.103), 6.67 (0.167) mm^2^ and 6.72 (0.197) mm^2^ in the amblyopic, fellow, and control eyes, respectively. The CC was significantly higher in amblyopic eyes than in fellow eyes (*P* = 0.014), but there was no significant difference between amblyopic and control eyes (*P* = 0.963).

### Choroidal thickness (CT)

The CT conformed to a normal distribution (*P* = 0.200). Within a 3 mm-diameter circle centered on the macular fovea, the mean CT was 388.6 ± 40.9 μm in the amblyopic eyes, 352.4 ± 56.6 μm in the fellow eyes, and 338.2 ± 70.2 μm in the control eyes (Table 2). The CT was significantly greater in amblyopic eyes than in fellow and control eyes (*P* < 0.001 and *P* = 0.006, respectively). After GEE analysis, the CT in the amblyopic eyes was significantly higher than that in the fellow eyes (*P* = 0.030) but was not significantly different from that in the control eyes (*P* = 0.160) (Table 3). There was a significant effect of axial length on CT; an increase in the AL was associated with an increase in the CT (*P* = 0.002). Age, sex, and BCVA had no effect on CT (Table 3).

**Table 2:** CT, 3D-CVI, and 3D-CVV in the amblyopia group and control group

	Group	CT (μm)	CC (mm^2^)	3D-CVI	3D-CVV (mm^3^)
		mean ± SD	median (IQR)	mean ± SD	mean ± SD
0–3 mm area	AE	388.6 ± 40.9	6.69 (0.103)	0.400 ± 0.064	1.099 ± 0.205
	FE	352.4 ± 56.6	6.67 (0.167)	0.385 ± 0.048	0.966 ± 0.187
	CE	338.2 ± 70.2	6.72 (0.197)	0.352 ± 0.087	0.864 ± 0.291
Amblyopic vs. fellow eyes^a^	t/z	5.022	-2.451	1.225	3.326
	P	< 0.001^∗^	0.014^∗^	0.234	0.003^∗^
Amblyopic vs. control eyes^a^	t/z	2.909	-0.047	2.101	3.085
	P	0.006^∗^	0.963	0.042^∗^	0.027^∗^

*CT* choroidal thickness, *CC* choroidal capillary flow area, *3D-CVI* three-dimensional choroidal vascularity index, *3D-CVV* three-dimensional choroidal vascularity volume, *AE* amblyopic eye, *FE* fellow eye, *CE* control eye

^a^t test, compared to amblyopic eyes

^∗^statistically significant (*P* < 0.05)

**Table 3:** Eye parameters and their relationships with choroidal thickness in the GEE model

	Group	CT (μm)	Beta^a^	95% CI^a^	P^a^
		mean ± SD			
0–3 mm area	AE	388.6 ± 40.9		Reference	
	FE	352.4 ± 56.6	-26.476	-50.325, -2.628	0.030^∗^
	CE	338.2 ± 70.2	-31.463	-75.394, 12.469	0.160
AL			-20.154	-22.019, -7.289	0.002^∗^
Age			-1.554	-12.964, 9.838	0.789
Sex			17.058	-14.728, 48.844	0.293
BCVA			-28.638	-94.414, 37.139	0.393

*CT* choroidal thickness, *AE* amblyopic eye, *FE* fellow eye, *CE* control eye, *AL* axial length, *BCVA* best-corrected visual acuity

^a^GEE adjusted for age, sex, axial length and BCVA

^∗^statistical significance (*P* < 0.05)

### 3D-CVI and 3D-CVV

In the examination of the choroidal vasculature, the 3D-CVI and 3D-CVV conformed to a normal distribution (*P* = 0.200). The mean 3D-CVI was 0.400 ± 0.064 in the amblyopic eyes, 0.385 ± 0.048 in the fellow eyes, and 0.352 ± 0.087 in the control eyes; only the amblyopic and control eyes demonstrated a significant difference (*P* = 0.042; amblyopic vs. fellow eyes, *P* = 0.234). The 3D-CVV in amblyopic eyes was significantly higher than that in fellow eyes (*P* = 0.003) and control eyes (*P* = 0.027) (Table 2).

GEEs were used to adjust for the effects of axial length, age, sex, and BCVA on the 3D-CVI and 3D-CVV. The 3D-CVI in amblyopic eyes was significantly higher than that in control eyes (*P* = 0.038) but was not significantly different from that in fellow eyes (*P* = 0.407) (Table 4). There was no effect of axial length, age, sex, or BCVA on the 3D-CVI. Regarding 3D-CVV, amblyopic eyes had significantly higher values than fellow and control eyes (*P* = 0.023 and *P* = 0.046, respectively) (Table 5). There was no effect of axial length, age, sex, or BCVA on 3D-CVV (Table 5).

**Table 4:** Eye parameters and their relationships with 3D-CVI in the GEE model

	Group	3D-CVI	Beta^a^	95% CI^a^	P^a^
		mean ± SD			
0–3 mm area	AE	0.400 ± 0.064		Reference	
	FE	0.385 ± 0.048	-0.013	-0.044, 0.18	0.407
	CE	0.352 ± 0.087	-0.054	-0.104, -0.003	0.038^∗^
AL			0.012	-0.004, 0.027	0.142
Age			-0.008	-0.021, 0.004	0.189
Sex			0.001	-0.032, 0.034	0.942
BCVA			0.013	-0.047, 0.073	0.679

*3D-CVI* three-dimensional choroidal vascularity index, *AE* amblyopic eye, *FE* fellow eye, *CE* control eye, *AL* axial length, *BCVA* best-corrected visual acuity

^a^GEE adjusted for age, sex, axial length and BCVA

^∗^statistical significance (*P* < 0.05)

**Table 5:** Eye parameters and their relationships with 3D-CVV in the GEE model

	Group	3D-CVV (mm^3^)	Beta^a^	95% CI^a^	P^a^
		mean ± SD			
0–3 mm area	AE	1.099 ± 0.205		Reference	
	FE	0.966 ± 0.187	-0.114	-0.226, -0.002	0.046^∗^
	CE	0.864 ± 0.291	-0.235	-0.399, -0.030	0.023^∗^
AL			-0.020	-0.079, 0.039	0.511
Age			-0.015	-0.057, 0.027	0.481
Sex			0.048	-0.330, 0.165	0.441
BCVA			-0.083	-0.330, 0.165	0.512

*3D-CVV* three-dimensional choroidal vascularity volume, *AE* amblyopic eye, *FE* fellow eye, *CE* control eye, *AL* axial length, *BCVA* best-corrected visual acuity

^a^GEE adjusted for age, sex, axial length and BCVA

^∗^statistical significance (*P* < 0.05)

## Discussion

The normal structure and function of the choroid plays an important role in the nutrition and thermoregulation of the retina. In recent years, there has been increasing focus on the role played by the choroid in various ophthalmic diseases [19, 20]. To clarify the effect of amblyopia on choroidal vascular structure, we conducted this study. We found that the choroidal capillary flow area was greater in amblyopic eyes than in fellow eyes but was not significantly different from that in control eyes. After correcting for age, sex, axial length, and BCVA using GEEs, the CT and 3D-CCV were greater in the amblyopic eyes than in the fellow eyes, while no significant differences were observed in the 3D-CVI. Amblyopia causes a thickening of the choroid and an increase in the flow area of choroidal capillaries and of large and medium vessels. Compared with the control eyes, amblyopic eyes had increased 3D-CVI and 3D-CVV but no significant differences in CT. Axial length remained the main causative effect of the changes relative to the control eyes, but the effect in amblyopia was not significant.

Changes in the choroidal structure of amblyopic eyes have been reported in numerous studies. A previous meta-analysis by Liu [21] yielded similar results to our study, with greater CT values observed in anisometropic amblyopic eyes than in fellow and control eyes, regardless of whether the axial length was matched. Some studies have reported thinner choroidal thickness in children with high myopic amblyopic eyes compared with high myopic eyes [22, 23]. Most of the subjects selected in previous studies were hyperopic or strabismic amblyopic children, suggesting that there are differences in choroidal structure changes in amblyopic eyes with different refractive types and that choroidal changes in amblyopic eyes may be due to a combination of amblyopia and refraction.

The study by Huang et al. [24] showed that the choroidal capillary flow area was higher in amblyopic eyes than in both fellow eyes and control eyes, similar to our findings. Terada’s [8] study found an increase in vascular perfusion area in amblyopic eyes compared to control eyes, which was also similar to the findings in our study. In recent years, an increasing number of studies have focused on refractive states and choroidal changes. Some studies have suggested that the choroid plays an important role in the process of emmetropization in children [25]. The choroid can adjust the position of the retina both by adjusting its thickness to the refractive state (choroidal modulation) and by releasing cytokines that regulate extracellular matrix remodeling in the sclera. In a study of patients with refractive parametric amblyopia treated with one year of refractive correction and/or masking, a decrease in the vascular component and an increase in the stromal component of the choroid in amblyopic eyes were observed after treatment, whereas no significant changes were observed in nonamblyopic eyes [26], suggesting that the effect of amblyopia on the choroid is associated with a delay in emmetropization.

Some studies have obtained different findings regarding the CVI. In the study by Baek et al. [27], which showed increased CVV in amblyopic eyes, the CVI was found to be higher in amblyopic eyes than in fellow eyes but was negatively correlated with CT, suggesting a relative decrease in choroidal vascularity and insufficient blood supply in amblyopic eyes. A similar conclusion was reached by Furundaoturan et al. [28], who found a decrease in the CVI and an increase in the stroma in patients with anisometropia amblyopia. The study by Cevher et al. [29] found a lower CVI in hyperopic anisometropia amblyopia eyes than in fellow and control eyes, suggesting that hyperopia affects choroidal structure. These studies, however, did not correct for the axial length and analyzed only a single layer of horizontal scans through the central macular sulcus that included only the blood vessels in a 750 μm or 1000 μm region. The differences in the image range of the scanning protocol could be the source of inconsistency between the conclusions drawn by that study and the present one.

In our study, before correction, the CT was higher in amblyopic eyes than in fellow and control eyes. After correction for axial length, age, sex and BCVA using the GEEs, the CT of the amblyopic eyes differed only from that of the fellow eyes and was not significantly different from that of the control eyes. Additionally, axial length significantly affected choroidal thickness (*P* = 0.002). Several previous studies have demonstrated [10, 11] that axial length has an effect on the choroid; specifically, there is a negative correlation between axial length and CT, with shorter axial lengths associated with thicker choroids. Therefore, it is necessary to correct the axial length in choroid-related studies to account for eyes with differences in axial length.

No relationship was identified between age and the choroidal vascular structure in our study. Some previous studies have shown that age also affects the choroid; a study by Xiong et al. [12], for example, showed that the CT in orthopic and mildly myopic children aged 6–19 years progressively decreased with age. Other studies have yielded different results, such as Read et al. [10], who showed a gradual thickening of the choroid with age during childhood. The CT is greater in children aged 10 to 12 years than in children aged 7–9 years and children aged 4–6 years. Zhou et al. [13] showed that CT, CVV, and choroidal stroma volume (CSV) all decreased with age in the macular subfovea region; however, the CVI was not affected by aging. In our study, no effect of age was found on the choroid because the patients were younger and had a relatively small age distribution.

The choroidal differences in amblyopia found in this study were mainly reflected with respect to the fellow eyes and not to the control eyes. Most likely due to the comparative study of both eyes from the same subjects, we were better able to control for the effects of age, sex, genetic and environmental factors. Vincent’s [30] study found that in refractive reference patients, differences in ocular biological parameters mainly originated in the posterior segment of the eye, such as vitreous cavity depth (VCD), axial length (AL), and CT. Patients showed similar characteristics in other ocular biometric parameters in both eyes, such as corneal curvature, anterior chamber depth (ACD), and crystalline lens power. Therefore, studies in patients with monocular amblyopia can better control for other unknown confounding factors and obtain relatively reliable conclusions.

There are limitations in our study. It is well-known that hyperopia affects the choroidal vascular structure. For instance, hyperopia causes thick choroids [16, 31]. To eliminate the effects of hyperopia on the choroid, it would be ideal to compare children with similar hyperopia grades (along with age- and sex-matched controls but with normal corrected visual acuity), but the mean refraction of the amblyopic eyes in our study was + 5.09 ± 2.01, and children with similar refraction but without amblyopia were difficult to find, so an ideal control group could not be found. To minimize the interference of refraction between different groups, the GEE equation was used in our study to correct for the effect of axial length age and sex on choroidal vascular parameters. The expectation is that the resulting results would yield the true effect of amblyopia on the choroid as much as possible.

The sample size of this study is small. This study included patients in different stages of treatment, which may have partially influenced the results. Some studies have shown that treatment in amblyopic eyes increases BCVA, decreases CVI, and increases stromal volume [26]. Further longitudinal studies are needed to clarify the effect of treatment on the choroid. Due to the need to conduct an SS-OCTA examination, only patients who could cooperate with the examination for acquiring high-quality images were included in this study. Some younger patients and those with poor visual acuity were excluded, which may have led to some degree of bias in the results. Therefore, future studies with larger populations and follow-up are needed to clarify the effects of amblyopia in the choroid.

In conclusion, in amblyopic eyes, the choroid was thicker and the capillary flow area and vascular volume were greater than those of normal eyes, which may be related to a delay in emmetropization. Future studies on amblyopia should focus on the mechanism underlying the causes of choroid changes in amblyopia. In the future, choroidal vascular changes may be used as a potential indicator for predicting and treating amblyopia.

## Data Availability

The data that support the findings of this study are available on reasonable request from the corresponding author [Liu Yang and Dehai Zhu]. The raw data are not publicly available due to [state restrictions, them containing information that could compromise research participant privacy/consent].
